# Highly Efficient Ion Manipulator for Tandem Ion Mobility Spectrometry: Exploring a Versatile Technique by a Study of Primary Alcohols

**DOI:** 10.1021/acs.analchem.2c05483

**Published:** 2023-04-24

**Authors:** Alexander Bohnhorst, Anne Zygmanowski, Yu Yin, Ansgar T. Kirk, Stefan Zimmermann

**Affiliations:** † Institute of Electrical Engineering and Measurement Technology, Department of Sensors and Measurement Technology, Leibniz University Hannover, Hannover 30167, Germany; ‡ ACKISION GmbH, Appelstr. 9A, Hannover 30167, Germany

## Abstract

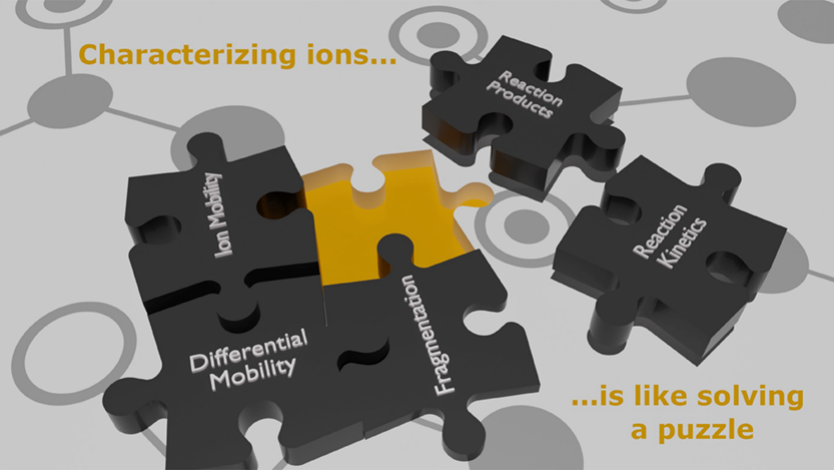

In this work, we present a tandem ion mobility spectrometer (IMS) utilizing a highly efficient ion manipulator allowing to store, manipulate, and analyze ions under high electric field strengths and controlled ion-neutral reactions at ambient conditions. The arrangement of tandem drift regions and an ion manipulator in a single drift tube allows a sequence of mobility selection of precursor ions, followed by storage and analysis, mobility separation, and detection of the resulting product ions. In this article, we present a journey exploring the capabilities of the present instrument by a study of eight different primary alcohols characterized at reduced electric field strengths *E*/*N* of up to 120 Td with a water vapor concentration ranging from 40 to 540 ppb. Under these conditions, protonated alcohol monomers up to a carbon number of nine could be dissociated, resulting in 18 different fragmented product ions in total. The fragmentation patterns revealed regularities, which can be used for assignment to the chemical class and improved classification of unknown substances. Furthermore, both the time spent in high electrical field strengths and the reaction time with water vapor can be tuned precisely, allowing the fragment distribution to be influenced. Thus, further information regarding the relations of the product ions can be gathered in a standalone drift tube IMS for the first time.

## Introduction

Ion mobility spectrometry (IMS) is an analytical technique separating ions in an electric field mainly by their size and shape.^1^ Utilizing highly efficient chemical ionization at atmospheric pressure, even the smallest concentrations of trace gases down to the ppt_v_ range and even below can be detected by IMS.^2^ There is a variety of different types of IMS,^3,4^ but particularly, drift tube IMS (DT-IMS) are used as standalone instruments for the detection of explosives,^5^ chemical warfare agents,^6^ toxic chemicals,^7^ and drugs of abuse.^8^ In a DT-IMS, ions are periodically injected into a drift region, where they are continuously accelerated by a constant electric field and decelerated by collisions. This leads to an average drift velocity *v*_D_, which is the product of the electric field strength *E* and ion mobility *K*. Thus, the ion mobility is always a property of the ion-neutral pair.^9^ The drift time *t*_D_ according to eq 1 is the time the ions need to reach the detector at the end of the drift length *L*_D_ after injection.
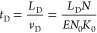
1

It can be expressed through Loschmidt’s number *N*_0_, which is the neutral density at standard conditions, the reduced ion mobility *K*_0_, which is also defined at standard conditions, and the reduced electric field strength *E*/*N* given in Td with 1 Td = 10^–17^ V cm^2^. It should be noted that *K*_0_ itself is a function of the reduced electric field strength *E*/*N* and other operational parameters as drift gas humidity. A detailed description and discussion of DT-IMS can be found elsewhere.^1^

A major challenge even for state-of-the-art ultra-high-resolution DT-IMS^10,11^ is the comparably low peak capacity compared to high-end analytical techniques such as mass spectrometry. Thus, a separation of ion species with similar *K*_0_ only by IMS is often difficult if not impossible. Even if the ion species can be separated, a reliable characterization or even identification is challenging due to the uncertainties caused by environmental influences on *K*_0_. Especially, the portable instruments required for the mentioned security applications suffer from low peak capacities and are typically used in rough and unpredictable environments. Thus, to prevent false negatives, wide alarm windows must be used, resulting in a potentially high rate of false positives as the density of values in the mobility separation space is high.^12,13^ In general, false positives occur if the spectrum contains peaks corresponding to nontarget substances but appearing within the required wide mobility window assigned to a target substance. While there is ongoing work to narrow the detection windows using mobility and instrument standards,^14−16^ these cannot account for all influences. To overcome this problem, various techniques have been presented in the literature. The most common solutions use hyphenated systems introducing another separation dimension, for example, a gas chromatograph (GC)^17^ combined with an IMS or an IMS combined with a mass spectrometer.^18,19^ However, these techniques suffer from long analysis times or high instrumental effort compared to a standalone DT-IMS. Another way to increase the analytical confidence of DT-IMS is acquiring ion characteristics that are orthogonal to *K*_0_. This can be achieved by a modification of the ion-neutral interaction in order to investigate other properties of the ions, e.g., binding energies or polarizability. Two common methods to modify the ion-neutral interaction are different drift gases,^20,21^ thus changing the ion-neutral interaction partners or manipulating the ion energy to influence the ion-neutral interaction itself. Imparting energy into the ion-neutral system can additionally influence the equilibrium of chemical reactions and lead to cluster dissociation or even fragmentation and thus to the formation of new ion species related to the target substance.^22^ IMS systems take advantage of increased ion energies including the high kinetic energy ion mobility spectrometer (HiKE-IMS) from the study of Langejürgen et al.,^23,24^ the tandem IMS with field-induced fragmentation from the study of Shokri et al.,^25−29^ and the field asymmetric time of flight IMS (FAT-IMS) from our group^30−32^ first published in 2015, which is the fundamental basis for this work. The ions’ energy, expressed as an effective temperature *T*_eff_, is according to the Wannier expression^33^ proportional to the square of the reduced electrical field strength *E*/*N*. Hence, instead of raising the temperature, an increased *T*_eff_ can simply be reached by increasing *E*/*N*. Thus, the HiKE-IMS is operated at a reduced pressure of about 20 mbar, leading to a low neutral density *N* similar to the reaction region of PTR-MS and allowing to establish a high *E*/*N* across both the reaction region and drift region with manageable voltages. Varying *E*/*N* in the reaction region allows other possible ionization reaction pathways for measuring hard-to-ionize substances and can enable field-induced fragmentation. Varying *E*/*N* in the drift region enables the acquisition of orthogonal information based on the field-dependent ion mobility, which can be described by the α-function. However, there are various reasons why operating IMS at ambient pressure is advantageous. No vacuum system is necessary and a higher pressure in the reaction region increases the efficiency of chemical ionization.^34−36^ To achieve effective temperatures up to 900 K at increased pressures, the drift field of the FAT-IMS was superimposed by a high-frequency rectangular electric field only in a small, spatially limited area—the FAT stage. In the FAT stage, ions could be shifted parallel to the drift direction to allow separation of overlapping peaks^30^ or improve compound identification by measuring both the low and high field mobility and additional product ions due to field-induced fragmentation.^31^ Shokri et al. expanded on this concept by combining multistage IMS separation with a fragmenter grid operated with a high-frequency sinusoidal electric field, allowing prior mobility selection of the ions to be fragmented.^25^ It could be shown that the mobility analysis of fragments enables the identification of chemical classes via IMS.^26^ Further improvements of design and operation methods results in the highly efficient ion manipulator presented in this work. In the ion manipulator, ions can be stored inside the ion manipulator for a few milliseconds while applying high electric field strengths and adding additional neutrals to induce chemical reactions. Building a multistage IMS with the proposed ion manipulator allows for increased efficiency and flexibility of ion manipulation, as both electric field and time parameters can be varied. In this manuscript, both the efficiency and the flexibility are demonstrated by a study of the fragmentation behavior of a homologous series of eight primary alcohols. This is an interesting substance class, e.g., in the field of online human metabolic^37^ and food flavor analysis.^38^

## Experimental Section

Our multistage IMS with tandem drift regions and a highly efficient ion manipulator in a single drift tube basically consists of the FAT-IMS which was expanded by a drift region and an additional ion gate prior to the ion manipulator. Here, we will focus on the features necessary for conducting the field-induced fragmentation and reaction experiments. All other instrumentation details will be covered in a second publication focusing exclusively on the instrumentation specifics.

**Figure 1:**
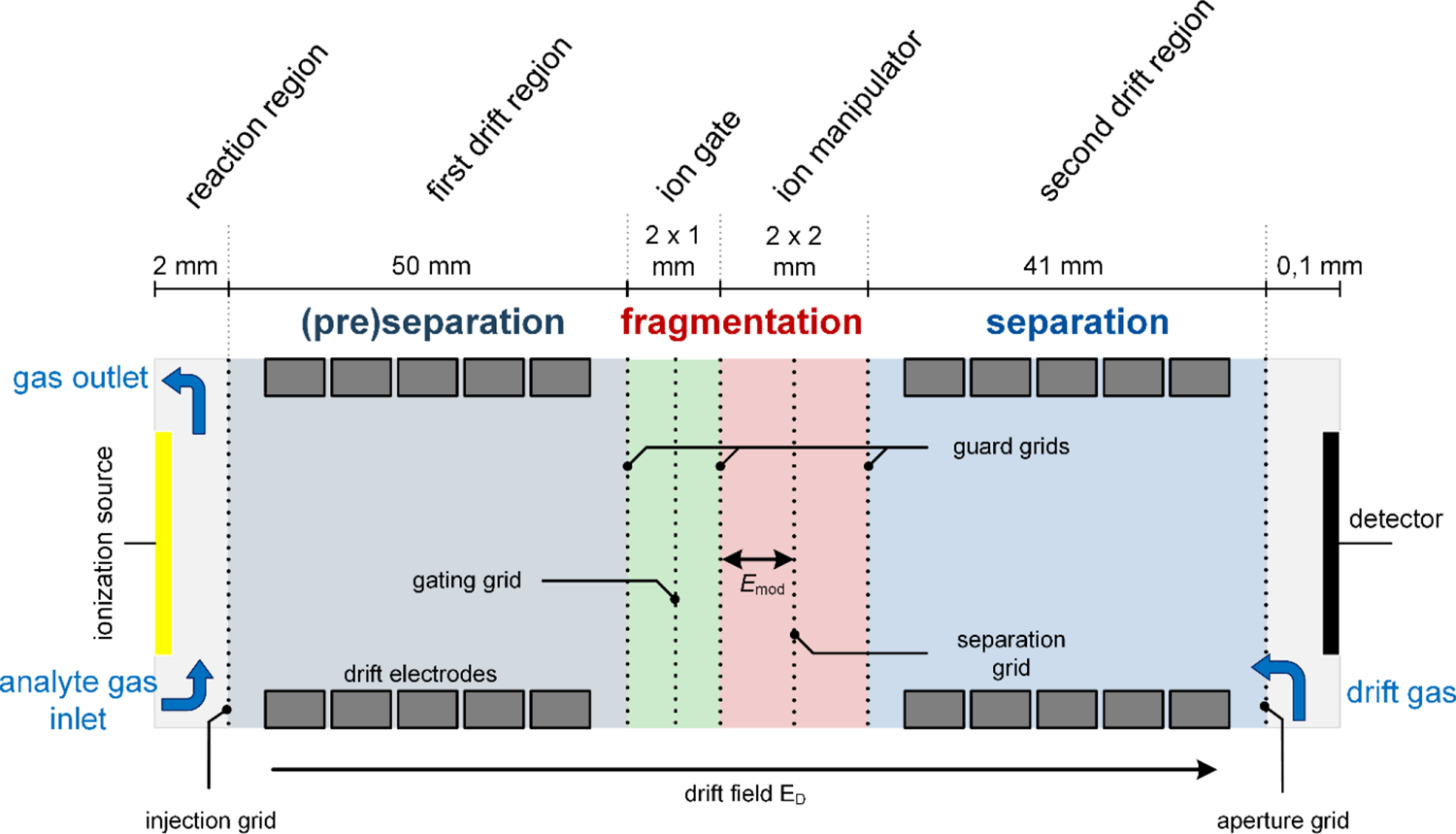
Schematic structure of the IMS with tandem drift regions and integrated ion manipulator. The device consists of six segments: The reaction region (left), a first drift region (gray), an ion gate prior to the ion manipulator (green), the ion manipulator (red), a second drift region (blue), and the ion detector (right).

Figure 1 shows a simplified schematic arrangement of the multistage IMS with tandem drift regions and ion manipulator consisting of six regions: the reaction region, where the analytes are ionized, the first drift region (gray), an ion gate (green), the ion manipulator (red), the second drift region (blue), and a Faraday detector shielded by an aperture grid. Spectra are acquired in two different modes: first, in the “IMS mode,” the electric fields in the ion gate and ion manipulator are adjusted to match the two drift fields, establishing a constant drift field across all four regions from the injection grid to the aperture grid. In this IMS mode, the resolving power is 110 and the recorded spectrum serves as the reference spectrum for the manipulation mode. In “manipulation mode,” ions are preseparated in the first drift region and gated into the ion manipulator, where the ions can be exposed to a defined *E*/*N* over a defined time. After ion manipulation, the resulting product ions are transferred into the second drift region and analyzed by their *K*_0_. The two modes, IMS mode and manipulation mode, are switched after every single spectrum, resulting in a repetition rate of 25 Hz for both spectra.

Protonated water clusters generated by a radioactive tritium ionization source^39^ in the reaction region serves as dominant reactant ions for ionizing the analytes supplied through the analyte gas inlet. A field switching shutter^40^ is used to gate the ions into the first drift region. Thus, ions are generated and accumulated in the nearly field-free reaction region for 20 ms and injected by switching the electrical potential of the ionization source. This type of ion shutter generates sharp ion clouds as necessary for operating the ion manipulator with minimal ion loss as the peaks must fit into the 2 mm gap between separation grids and adjacent guard grids. The drift regions consist of a series of concentrically arranged stainless-steel rings with an inner diameter of 21 mm, enclosed by a housing made of polyetheretherketone (PEEK).^10^ The first drift region has a length of 50 mm (injection grid to first guard grid), and the second drift region has a length of 41 mm (last guard grid to aperture grid). The ion manipulator is formed by three consecutive grids, forming two regions, each with a length of 2 mm. The modification field is generated by varying the potential of the separation grid while the two adjacent guard grids separating the drift regions and the ion manipulator prevent the modification field from penetrating into the drift regions. All grids are made of etched 100 μm thick stainless-steel foils with a hexagonal structure.^11,40^ The optical transparency is 0.6 for the injection grid, 0.7 for the aperture grid, and 0.8 for the gating grid separation grid and guard grids. The field switching shutter is supplied by a self-build isolated and switchable power supply with an output voltage of up to 500 V. The voltages for the two resistive voltage dividers supplying the drift regions are generated by self-build isolated high voltage supplies with an output voltage of up to 5 kV. The high voltage for the separation grid is supplied by an HCP 35-12500 (FuG Elektronik GmbH, Germany) with an output voltage of up to 12.5 kV. The asymmetric rectangular waveform at the separation grid is generated by switching the output voltage of this high voltage supply with an HTS 91-01-HB-C HFB LP solid-state switch (Behlke Power Electronics, Germany).

In manipulation mode, the drift fields in the ion manipulator are switched off exactly when the ions are in the ion manipulator—between the separation grid and the second guard grid. Thus, the drift movement of the ions is stopped, which allows storing the ions for an adjustable storing time *t*_stor_ as long as the drift fields are switched off. During this storing step, ions located in the ion manipulator can be exposed to an alternating electrical field, the modification field, or undergo chemical reactions with neutrals.

**Figure 2:**
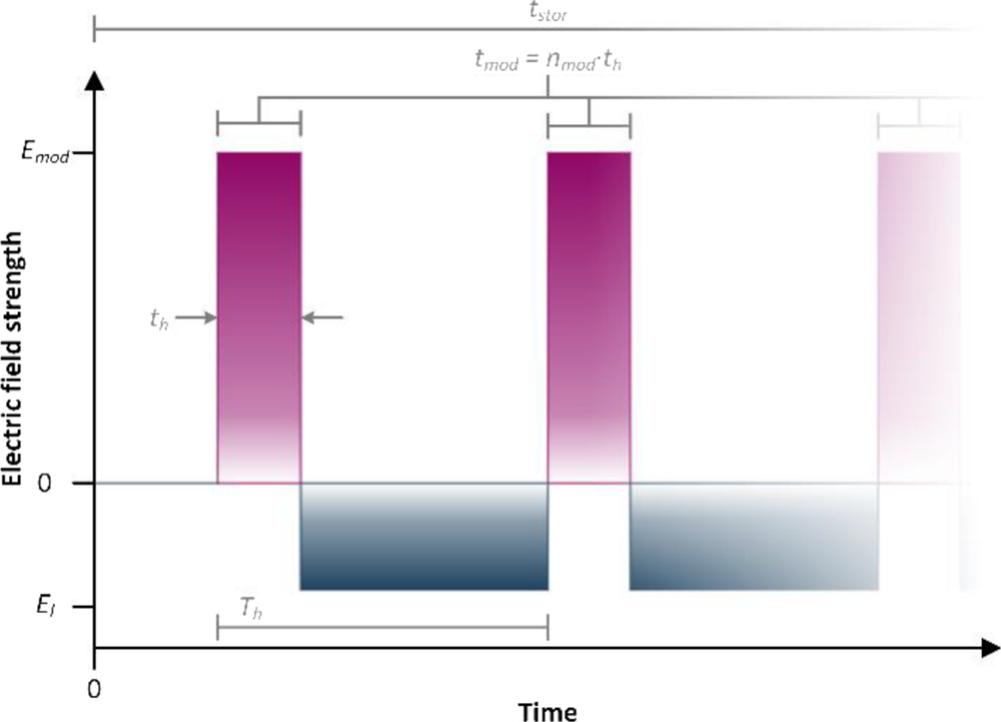
Asymmetric waveform used for ion manipulation. The mean of the waveform is chosen to minimize the ions’ net movement during the modification. Storing the ions in a field-free region prior or after the modification field is applied offers additional analytical possibilities.

An example for the waveform of the modification field is shown in Figure 2. This waveform consists of a variable number of short pulses with a high electrical field strength followed by long intervals with a low electric field strength, but the opposite sign. Thus, the modification field utilized in our setup is defined by the modification field strength during the high field pulse *E*_mod_, the field between two pulses *E*_l_, the number *n*_mod_ and width *t*_h_ of the high field pulses, and the period *T*_h_ of one full cycle of high and low field interval.

During all measurements, the pressure in the drift regions and the ion manipulator was kept at ambient pressure. Thus, to vary *E*_mod_ from 0 to 120 Td, the potential of the separation grid relative to the guard grids was adjusted from 0 to 6500 V. The field strength during the low field period *E*_l_ was adjusted accordingly to minimize the net ion movement during manipulation. The modification time *t*_mod_ is defined as the time the ions are exposed to the high field conditions. It may be calculated by multiplying cycle number *n*_mod_ and pulse width *t*_h_. As the ions’ net movement is close to zero during the manipulation, losses due to ions being discharged on the separation grid or guard grids are minimal even for high storing times. Hence, adjusting *t*_mod_ is possible by simply increasing the number of waveform cycles *n*_mod_. In this setup, modification times of up to 50 μs are possible.

As storing and exposing the ions to the modification field are two independent steps, the ions may be stored for a given time prior or after modification. This allows the reaction of precursor or product ions of the modification and neutrals located in the ion manipulator, enabling further analytic possibilities. Thus, this design offers control over ion temperature by varying *E*/*N*, control over the modification time *t*_mod_ by increasing *n*_mod_ and control over the reaction time with neutrals due to an optional storing step. It should be noted that product ions generated in the ion manipulator and neutrals in the drift gas can undergo chemical reactions during the drift time *t*_d,2_ in the second drift region. Hence, the overall reaction time is *t*_reac_ = *t*_stor_ + *t*_d,2_. Thus, *t*_reac_ can be increased by simply increasing *t*_stor_ or by increasing *t*_d,2_ due to a reduced drift field. Logically, *t*_d,2_ is the lower bound of *t*_reac_. Table 1 summarizes the operational parameters of the device.

**Table 1:** Operational Parameters of the IMS with Tandem Drift Regions and Integrated Ion Manipulator

parameter	value
tritium source activity	160 MBq
reaction region length	2 mm
reaction region injection field	10 Td (250 V/mm)
reaction region blocking field	0.01 Td (0.25 V/mm)
injection time	200 μs
first drift region length	5 mm
second drift region length	41 mm
drift region field *E*_D_/*N*	3–4.5 Td (75–115 V/mm)
manipulator region length	2 × 2 mm
modification field *E*_mod_/*N*	0–120 Td (0–3000 V/mm)
high field pulse width *t*_h_	0.5 μs
high field pulse cycle period *T*_h_	50 μs (20 kHz)
modification time *t*_mod_	0–50 μs
storage time *t*_stor_	0–4 ms
reaction time *t*_reac_	1.5–8 ms
injection grid transparency	0.6
separation grid transparency	0.8
guard grid transparency	0.8
aperture grid transparency	0.7
resolving power (reactant ions)	110
repetition rate	25 Hz (full cycle of both modes)
drift gas and carrier gas	dry and purified air
drift gas flow	150 mls/min^a^
sample gas flow	20 mls/min^a^
operating temperature	20–24 °C
operating pressure	998–1015 mbar
dew point drift/sample	–85 to −100 °C

a Milliliter standard (mls) per minute, mass flow at reference conditions 20 °C and 1013.25 mbar.

The dissociation of eight different alcohols up to a carbon chain length of 9 was investigated. Samples of 1-ethanol, 1-propanol, 1-butanol, 1-pentanol, 1-hexanol, 1-heptanol, 1-octanol, and 1-nonanol were purchased from Sigma-Aldrich (MI, USA) in the highest available purity. Details regarding the gas mixing system can be found in the previous publication.^30^ For each substance, the protonated monomer was mobility-selected, isolated, and used as a precursor for the following ion manipulation. The reduced modification field strength *E*/*N* was varied from 20 to 120 Td in 2 Td steps five times over a period of 4 h to evaluate the average value of the overall charge for each ion species present in the spectrum and the standard deviations of the measured values. All spectra shown were captured with an averaging time of 50 s. For the evaluation of charge and mobility, the peaks were fitted with a Gaussian function in MATLAB 2020 (The MathWorks, Inc., MA, USA). The position, height, and width of the fitted peaks are indicated in the figures shown. This is important to keep in mind, as even with a constant number of ions and thus constant charge, the peak amplitude depends on the peak width and thus on the mobility. A simple evaluation of peak amplitudes can therefore lead to significant misinterpretation of the experimental data. 2,6-Di-*tert*-butylpyridin (*K*_0, std_ = 1.417 cm^2^/V s)^41,42^ is used as a mobility standard in all measurements. Ion mobilities of precursors and product ions are referenced to this mobility standard. While the values stated in the figures and in Table 3 are given as reduced mobility, the mobility axis in the spectra is given in V s/cm^2^, which is the inverse reduced mobility. This is useful as the inverse mobility is proportional to the collision cross section^1^ and hence offers a better measure when product ions are compared. It should be noted that the ions formed in the ion manipulator are separated only in the second drift region. Accordingly, the product ions’ mobilities are calculated based on the drift time and the electrical field in the second drift region. Thus, the mobility axis in manipulation mode is stretched compared to the one in IMS mode, which results in seemingly broadened peaks with a resolving power of roughly 40. Especially, it should be noted that the peak areas in the plots analyzing IMS mode and manipulation mode are not comparable due to the differently stretched mobility axis. A more detailed explanation of the algorithms used for the data analysis will be found in the instrumentation publication.

## Results and Discussion

The protonated alcohols in the first drift region of the IMS can be represented by the molecular formula [ROH]H^+^ · H_2_O with the carbon chain R and the alcohol functional group OH. In the case of primary alcohols, R can be fully described by the carbon number of the chain *n*_c_, resulting in the formula C_*n*_c__H_(2*n*_c_ + 1)_. In the context of an IMS operated under atmospheric pressure, further water molecules may form a cluster with the protonated alcohol molecules. However, these are not shown here for the sake of simplicity. The literature describes various fragmentation mechanisms when high kinetic energies are applied to this type of ions. At moderate energies, the water cluster size is reduced^24,43,44^ due to dehydration until the protonated monomer is present.
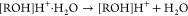


A further increase in the applied energy can lead to various fragmentation processes. Four important mechanisms can be identified in the literature. Particularly with low carbon numbers, the cleavage of molecular hydrogen resulting in the carboxy ion RO^+^ can be observed (a).
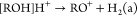


This is often followed by a cleavage between the carbon chain and the oxygen. Thus, the alcohol fragments into a neutral water molecule and the carbocation R^+^(b).
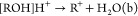


This may be followed by two different reactions. Either hydrogen abstraction occurs, a process in which molecular hydrogen is released by the formation of a double bond in the carbon chain (c)
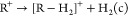
or the carbon number is reduced due to the cleavage of a C–C bond, forming a CH_4_ molecule (d).
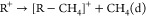


However, the abstraction of CH_4_ requires a high activation energy. Furthermore, only alcohols with a carbon number *n*_c_ equal to or higher than 4 tend to show this fragmentation path.^45−47^ As alcohols with a higher carbon number have lower mobilities, the field strength applied in the ion manipulator might not be sufficient to reach the activation energy. Therefore, the occurrence of the fragmentation mechanism (d) in our ion manipulator is rather unlikely.

In this work, a total of 8 different protonated alcohols were investigated. Their fragmentation resulted in the formation of up to four additional product ions. In total, in addition to the 8 precursors, 18 different product ions could be observed. A reliable identification of all product ions would require a mass spectrometer following the IMS. However, the transfer into a mass spectrometer could influence the structure of the product ions and would not be feasible for field use. Therefore, we believe that strategies to achieve a certain degree of identification only by utilizing the possibilities of an IMS equipped with an ion manipulator are of great interest for both fundamental understanding and chemical detection. In a first step, we investigate the fragment patterns of individual precursors and demonstrate the impact of parameters such as the modification field strength *E*_mod_/*N*, the modification time *t*_mod_, and the reaction time *t*_reac_. Furthermore, the influence of water as a “modifier” in the drift gas is also studied. This allows building a reaction scheme showing the correlations between the different product ions of a given precursor. In a second step, global trends are used to sort precursor and product ions into clusters and, if possible, make assumptions about the properties of these clusters. These two strategies will help us to piece together our puzzle of fragments into one picture.

### Variation of the Modification Field *E*_mod_

Starting out, we will focus on 1-butanol as a first example compound. Due to the carbon number of 4 and the high mobility, the occurrence of a CH_4_ cleavage is most likely here. Furthermore, detailed descriptions of the fragmentation characteristics of 1-butanol are already available in the literature, which allows a comparison of the results obtained by different systems. Thus, 1-butanol seems to be a good choice to characterize our instrument. The first parameter to be evaluated is the influence of the modification field strength *E*_mod_/*N*, which is a measure for the energy as stated by the Wannier expression^33^ and therefore affects the composition and intensities of the product ions.

**Figure 3:**
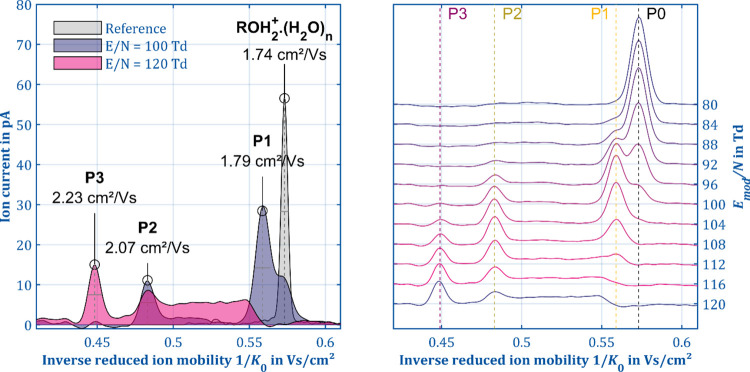
Reference spectrum in IMS mode (gray) and the spectra in ion manipulation mode with *E*_mod_ = 100 Td (blue) and 120 Td (purple) of 3 ppb 1-butanol (left). Comparison of the spectra for *E*_mod_ from 80 to 120 Td (*E*_D_ = 3 Td, *n*_mod_ = 5, *t*_mod_ = 2.5 μs, *t*_reac_ = 2.48 ms, *T* = 298 K, *P* = 1008 mbar).

Figure 3 shows the ion mobility spectrum of 1-butanol in IMS mode (gray) and manipulation mode at a modification field strength of 100 Td (purple) and 120 Td (blue). In the IMS mode, both the protonated monomer (ROH · H_3_O^+^,1.74 cm^2^/V s) as well as the proton-bound dimer ([ROH]_2_H^+^,1.47 cm^2^/V s, not shown in Figure 3) appear as baseline-separated peaks. The absence of other product ions due to an in-source fragmentation can be attributed to the soft ionization via hydrated reactant ions.^48^ Utilizing a tandem IMS, Shokri et al. reported two product ions with a mobility of 2.01 and 1.81 cm^2^/V s at an *E*/*N* of 129 Td.^26^ Utilizing a HiKE-IMS, Weiss et al. reported the formation of the three product ions RO^+^, R^+^, and [R – H_2_]^+^ at an *E*/*N* of 115 Td.^45^ However, in manipulation mode, a total of three different product ion species (P1–P3) and the reaction product B3↑ are formed. The task is now to utilize the capabilities of the ion manipulator, to gather more data, and to finally assign specific fragments according to the reactions (a)–(d). The intensities of the individual ion species strongly depend on *E*_mod_/*N*, which agrees well with the results reported in the literature by HiKE-IMS and PTR-MS experiments.^45,46^ At 100 Td, the protonated 1-butanol as precursor P0 is still present next to the two product ion species P1 (*K*_0_ = 1.79 cm^2^/V s) and P2 (*K*_0_ = 2.07 cm^2^/V s). P1 and P2 arise as two baseline-separated peaks, but P1 and P0 exhibit similar mobilities. Thus, P1 and P0 could not be completely separated in the second drift region. However, fitting both peaks with overlapping Gaussian curves to evaluate peak positions and intensities is feasible since one can expect just two peaks of the Gaussian shape. Increasing the field strength to 120 Td causes three phenomena. First, the third product ion species P3 (*K*_0_ = 2.23 cm^2^/V s) appears. Second, both the precursor P0 and the product P1 are completely dissociated and the intensity of P2 is slightly reduced. Third, the baseline in the mobility interval between P1 and P3 is raised. The baseline current gradually increases to 8 pA and then abruptly drops to 0 pA, indicating a fourth ion species B3↑ with mobility between P3 and P0, somewhere close to P1.

This fourth ion species seems to be the product of a reaction between P3 and the neutral components of the drift gas in the second drift region. To our knowledge, this is the first time this behavior of the alcohol fragments is observed in an IMS. The slope of the raised baseline offers information about the direction of the reaction. Since the population of the educt is high at the beginning of the second drift region, immediately after ion manipulation, the reaction rate and thus the population of the reaction product is the highest here. At the end of the drift region, the population of the educt is lower due to the continuous depletion, and consequently, the population of the product ions decreases. The result is a current gradient in the ion mobility spectrum, which increases when approaching the ion mobility of the reaction product. Following, this unknown ion species is referred to B3↑ if the baseline’s gradient is positive approaching P0 and B3↓ if the gradient is negative. Thus, the origin and direction of the reaction are reflected. From the fact that the reaction takes place in the second drift region, two points emerge. First, the reaction time *t*_reac_ is roughly given by the time the ions spend in the ion manipulator and the time the ions need to pass the second drift region reaching the detector. Second, as the ion species is formed in the drift region, the ion mobility measurement is difficult. Thus, it is nearly impossible to predict which ion species is involved in B3↑ only based on the measured ion mobility. However, the sharp drop of the baseline at about 1.81 cm^2^/V s, which is approximately the peak position of P1, suggests that the reaction product B3↑ could be P1. However, further studies would be necessary to be confident.

**Figure 4:**
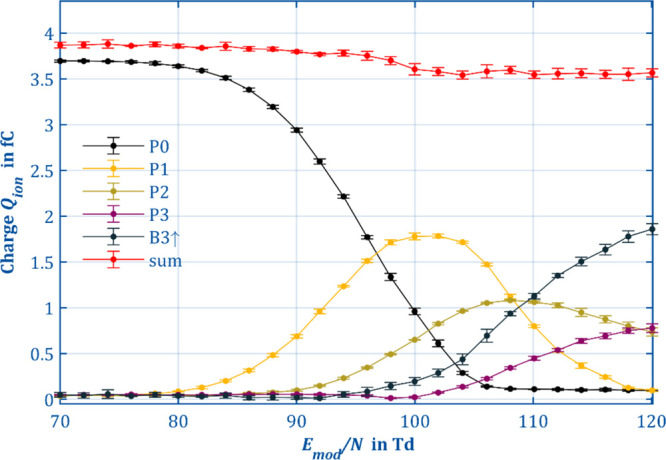
Total charge in averaged IMS spectrum of the precursor 1-butanol monomer (black), the first product ion species P1 (yellow), the second product ion species P2 (green), the third product ion species P3 (purple), the baseline increase between the first and third product ion species B3↑ (teal), and the sum of the charge (red) in relation to the separation field strength (*E*_D_ = 3 Td, *n*_mod_ = 5, *t*_mod_ = 2.5 μs, *t*_reac_ = 2.48 ms, *T* = 298 K, *P* = 1008 mbar).

To gain a better understanding of the relationships between the four ion species P0, P1, P2, P3, and B3↑, the charge corresponding to the individual product ions is plotted against the reduced modification field strength *E*_mod_. Thus, the curves in Figure 4 allow several observations. First, according to the total charge (red), which is the sum of all individual charges, ion losses in the ion manipulator are quite low. Only 8% is lost when comparing the charge of 3.86 fC at 80 Td to 3.54 fC at 120 Td. It is noteworthy that the losses occur almost only when P0 fragments to P1. As soon as P0 is completely depleted, the total charge remains constant. A constant total charge is greatly helpful for quantitative observations, and it indicates the quality of the fit model as the charge present in the spectrum is fully explained.

Figure 4 supports the assumption that P0 fragments to P1 since the intensity of P1 increases as long as P0 decreases. Beyond 90 Td, P1 starts to react further to P2. Thus, the intensity of P1 reaches a maximum at 102 Td. Thereafter, P0 is almost completely depleted, stopping additional formation of P1. The same can be seen for P2, which reacts to P3 starting at about 96 Td and reaches its maximum intensity at 108 Td, just before P1 is almost completely depleted. A direct fragmentation P0 → P3 or P1 → P3 seems unlikely as the charge of P3 increases even when P0 and P1 are depleted but cannot be excluded with absolute certainty. The charge in the raised baseline B3↑, which indicates a reaction in the second drift region, increases as P3 is formed, again indicating the presence of a reaction product of P3.

**Figure 5:**
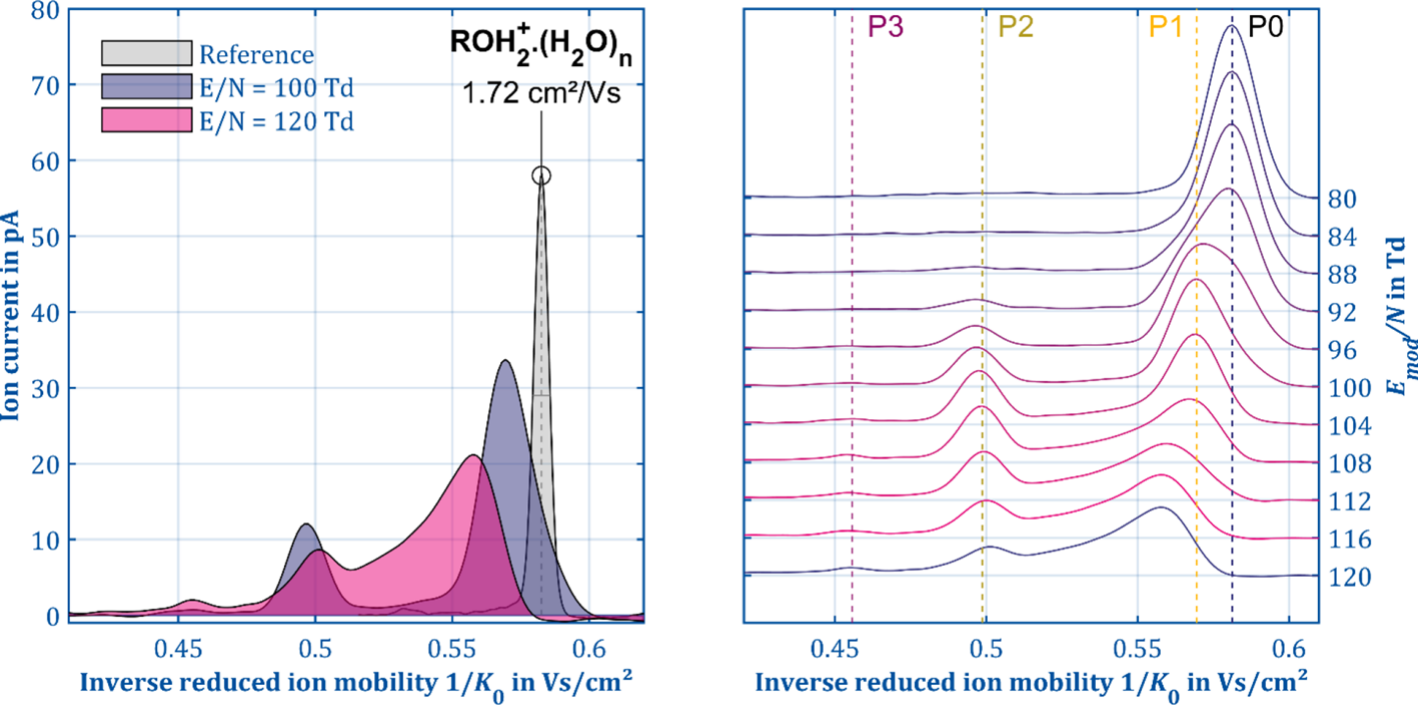
Reference spectrum in IMS mode (gray) and the spectra in ion manipulation mode with *E*_mod_ = 100 Td (purple) and 120 Td (blue) of 3 ppb 1-butanol (left) with a water concentration of 540 ppb in the drift gas. In IMS mode, only a small shift in the ion mobility of the protonated monomer can be observed compared to the dry conditions. In ion manipulation mode, the intensity distribution of the product ions drastically changes as can be seen in the right panel (*E*_D_/*N* = 4.5 Td, *n*_mod_ = 5, *t*_mod_ = 2.5 μs, *t*_reac_ = 1.93 ms, *T* = 298 K, *P* = 1008 mbar).

Thus, two questions remain: which of the fragmentation mechanisms (a)–(d) listed above are occurring here and which molecule is the neutral reaction partner of P3. The latter can be tested by changing the composition of the drift gas. For this purpose, the water vapor concentration in the second drift region was increased from 40 ppb for the dry conditions to 540 ppb for more humid conditions. The spectra captured in manipulation mode (Figure 5) reveal significant differences to the spectra obtained under dry conditions. While the moisture has only a small influence on P1 and P2, the intensity of P3 decreases drastically and the charge of B3↑ increases in turn.

**Figure 6:**
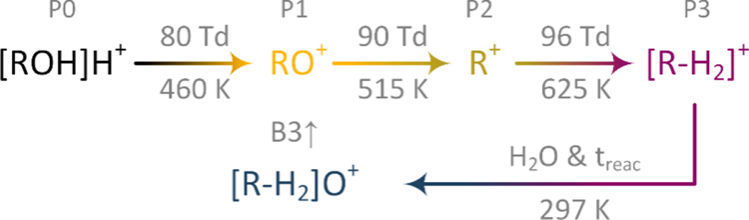
Assumed reaction pathways of the protonated monomer of 1-butanol (P0). 1-Butanol seems to fragment in three steps by sequential cleavage of hydrogen (P1, RO^+^), oxygen (P2, R^+^), and hydrogen (P3, [R–H_2_]^+^). The effective temperatures when the activation energy of the fragmentation is reached are calculated according to the Wannier expression.^33^ After fragmentation, a reaction of the product ions with neutral water could be observed, leading to a fourth product ion (B3↑, [R–H_2_]^+^·H_2_O).

In a nutshell, based on the results presented by Weiss et al.,^45^ P1 can be assumed to be RO^+^. P1 is highly unlikely to be the dehydrated protonated monomer as hydration could not be observed even under high humidity conditions. Further following the reaction scheme from Weiss et al., P2 should be the carbocation R^+^ and P3 the product of hydrogen abstraction [R – H_2_]^+^. Again, the carbocation R^+^ is stable even under high humidity conditions,^49,50^ but the measurement shown in Figure 5 is a strong indicator for a reaction of P3 and neutral water molecules in the second drift region. With an estimated mobility of 1.81 cm^2^/V s, similar to the protonated monomer, B3↑ could be the butyryl cation [R – H_2_]O^+^.^48^ The presumed reaction pathways of 1-butanol are shown schematically in Figure 6.

However, while this scheme seems reasonable, it raises the question why these results differ from the results obtained by the tandem IMS utilized by Shokri et al. In the study presented there, only two product ions^26^ are reported for the protonated 1-butanol, while in this work, four different product ion species could be observed. Despite different experimental parameters such as modification times and water vapor concentrations, these differences could also be attributed to the higher resolving power achieved with our device. For a baseline separation of P0 and P1, a resolving power of at least 60 is needed.^51^ Thus, even the drift tube utilized in our setup is barely sufficient for detection. This is a valuable finding as it demonstrates the importance of high-resolution IMS even in setups utilizing orthogonal separation techniques and emphasizes the potential of ongoing research and improved instrumentation in the field of IMS.

### Variation of the Modification Time *t*_mod_

**Figure 7:**
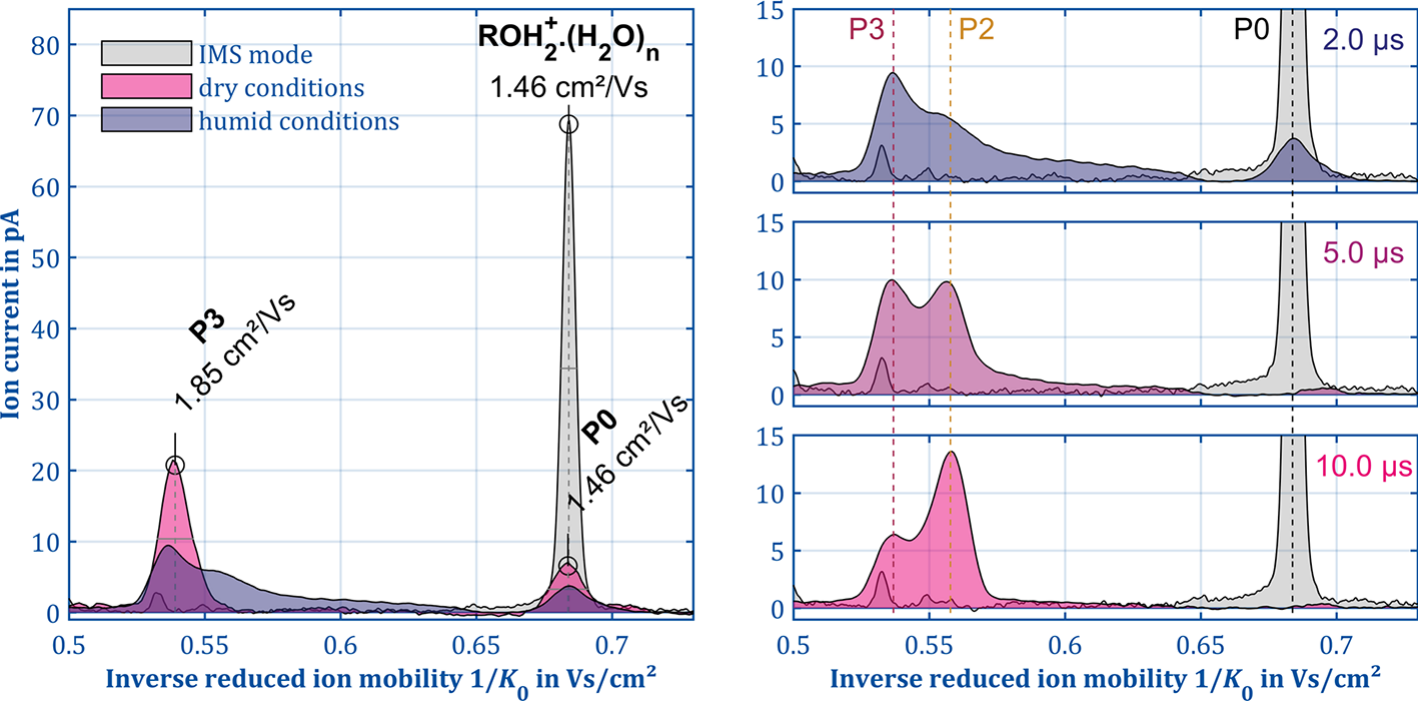
Reference spectrum in IMS mode (gray) and the spectra in ion manipulation mode for dry conditions (yellow) and with an increased water vapor concentration (purple) of 80 ppb 1-heptanol (left). Increasing the water vapor concentration and the modification time *t*_mod_ (right) from 2 to 10 μs leads to the formation of two additional product ions (P2 and B3↓) and changes the product ions intensities in favor of P2. It should be noted that impurities visible in IMS mode are filtered out in ion manipulation mode (*E*_D_/*N* = 3 Td, *E*_mod_/*N* = 120 Td, *t*_mod_ = 2–10 μs, *t*_reac_ = 3.7 ms, *T* = 297 K, *P* = 1010 mbar).

A completely different situation can be seen when investigating alcohols with a higher carbon number, such as 1-heptanol. The ion mobility spectrum of 1-heptanol in IMS mode shown in Figure 7 (left) contains the protonated monomer (ROH · H_3_O^+^,1.46 cm^2^/V s) and the proton-bound dimer ([ROH]_2_H^+^,0.86 cm^2^/V s, the latter not shown in Figure 7). In contrast to the observations found for 1-butanol, where three product ions appeared, only one product ion (P3, *K*_0_ = 1.85 cm^2^/V s) is formed under the same dry conditions with the protonated 1-heptanol monomer as the precursor ion (yellow). This seems plausible as the ion mobility of P0 of 1-heptanol is about 16% lower than that of 1-butanol, which leads, according to the Wannier expression,^33^ to a 30% reduced energy even when utilizing the same modification field *E*_mod_/*N*. Therefore, it can be expected that both the species and the intensities of the product ions differ. However, if P3 is the product of the same fragmentation mechanism for 1-butanol and 1-heptanol, a reaction with the neutral water molecules of the drift gas would again be expected. Indeed, the peak shape of P3 is not Gaussian but slightly asymmetric, indicating the existence of a reaction. However, the effect on the spectrum is significantly smaller than that in the case of 1-butanol, and interestingly, the reaction equilibrium seems to be reversed. Again, it is possible to influence the reaction by increasing the water concentration in the drift region. In the resulting spectrum (purple), the asymmetry of the peak of P3 increases so that an increase of the baseline, similar to that of 1-butanol, is formed, almost completely covering the interval between P3 and P0. Two details are of particular interest. First, the slope of the baseline is reversed compared to Figure 5. Thus, unlike 1-butanol, P3 is the product and not the educt of the reaction. Second, a hump of the baseline reveals another product ion P2, which might be the carbocation R^+^ analogues to the fragmentation pattern of 1-butanol.

Thanks to the design of the ion manipulator, it is possible to vary the modification time *t*_mod_—i.e., the time during which the ions are exposed to the high electric field strengths—independently from the modification field *E*_mod_. This is a major advantage compared to, for example, HiKE-IMS, PTR-MS, or the tandem IMS used by Shokri et al. This feature proves particularly useful in case of 1-heptanol, as demonstrated in the right panel of Figure 7. Here, spectra for three different *t*_mod_ ranging from 2 to 10 μs are shown. Even in this small interval of only 8 μs, the dominant ion species completely changes. For modification times <2 μs, P0 is still present in the spectrum and P3 is the dominant peak while P2 can hardly be detected. Increasing *t*_mod_ to 5 μs leads to a complete dissociation of P0 while P2 and P3 are now showing similar intensities. Again doubling *t*_mod_ to 10 μs increases the intensity of P2 to about twice the intensity of P3. This means that in the case of 1-heptanol, P2 is not a secondary product of P1 as shown by the observation of 1-butanol. This suggests that it must be a different ion species. Hence, a new reaction scheme is needed to for 1-heptanol describing the observed behavior. To figure out the origin of P2, the capabilities of the ion manipulator can be utilized again.

### Variation of the Reaction Time *t*_reac_

**Figure 8:**
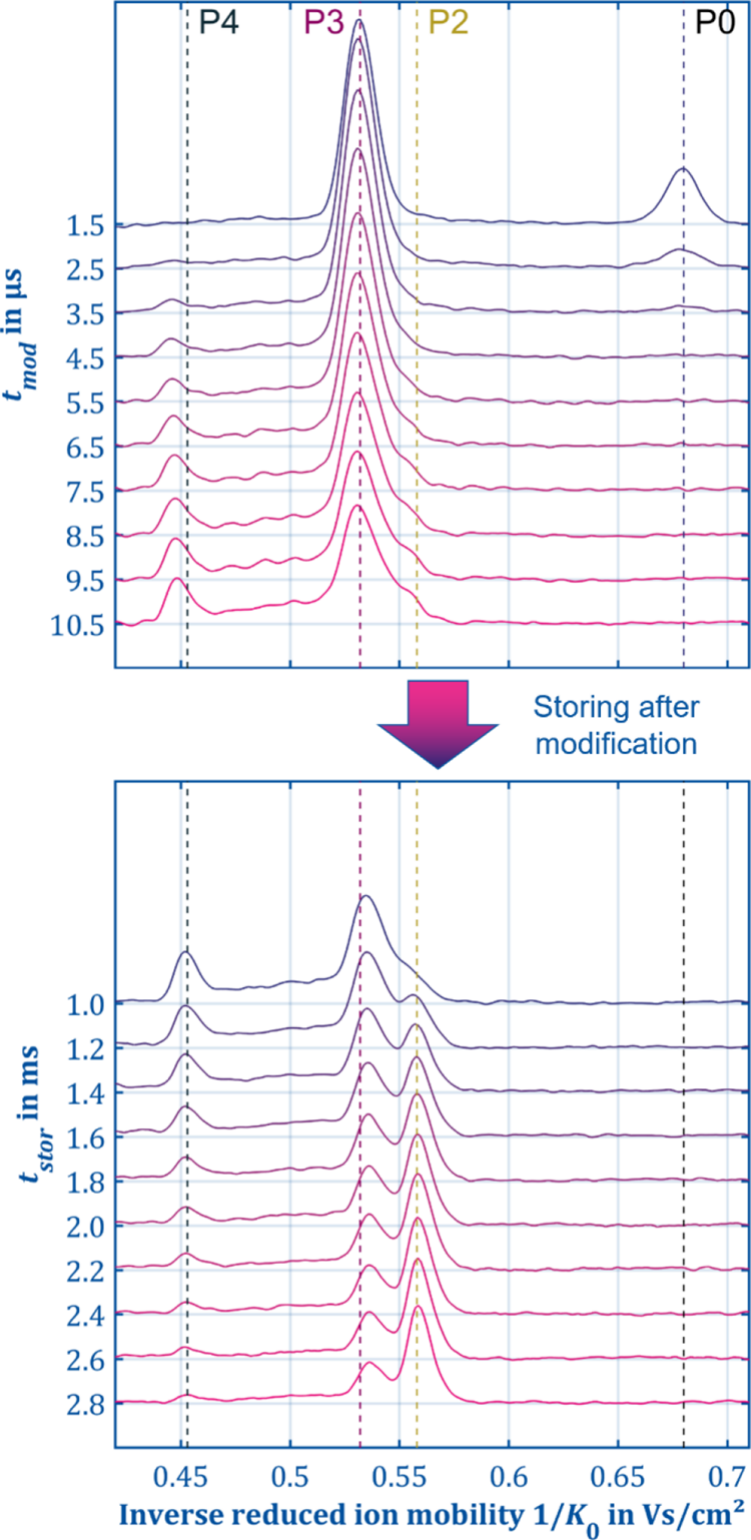
Spectra in ion manipulation mode for dry conditions (240 ppb water vapor concentration) of 80 ppb 1-heptanol with increasing *t*_mod_ (top) without additional storage and spectra at a constant *t*_mod_ of 10.5 μs with an increased storage time *t*_stor_ up to 3 ms (bottom). This experiment shows how P3 fragments further to P4, which then reacts to P2 (*E*_D2_/*N* = 4.5 Td, *E*_mod_/*N* = 120 Td, *t*_reac_ = 1.9–4.9 ms, *T* = 296 K, *P* = 1015 mbar).

**Figure 9:**
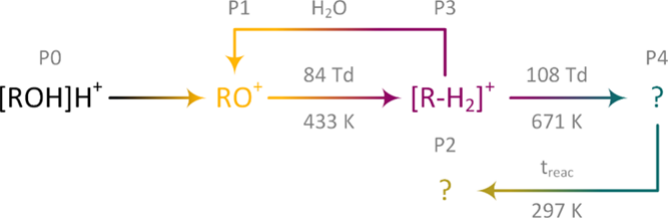
Assumed reaction pathways of the protonated monomer of 1-heptanol (P0). 1-Heptanol shows four product ions, where P3 and P4 seem to be the products of a fragmentation of P1. Thus, it is not a linear reaction as observed for 1-butanol.

For this purpose, the humidity in the drift region is lowered again to about 230 ppb and the reaction time *t*_reac_ is reduced to 1.9 ms by increasing the drift field in the second drift region from 3 to 4.5 Td. Thus, only P3 is present at *E*_mod_/*N* = 120 Td in the manipulator mode. Increasing the modification time *t*_mod_ again from 1.5 to 10.5 μs (Figure 8, left) now reveals a completely new result. In contrast to the elevated humidity conditions in Figure 7, P3 does not directly turn into P2, but another fragment P4 is formed with a surprisingly high reduced ion mobility of about 2.2 cm^2^/V s which might be a result from a α-cleavage, resulting in the formation of COH_3_^+^. Moreover, a new reaction product can be observed which seems to originate from P4. Extending the analysis in the high field at *t*_mod_ = 10.5 μs by an additional storage of the ions from *t*_stor_ = 1 to 3 ms (Figure 8, right) now shows very impressively the origin of P2, which is the reaction of P4, and makes it possible to extend the reaction scheme of 1-heptanol shown in Figure 9. Thus, to put it in a nutshell, P2 is not formed from P1 but from the reaction of P4 with neutral water. Instead, P3 seems to be a fragment of P1 which reacts with neutral water in the drift tube back to P1. Increasing *t*_mod_ leads to a depletion of P3 as it fragments to P4. Thus, less P3 enters the second drift region, resulting in a lower intensity of P1, but higher intensities of P4 which further reacts to P2 if *t*_reac_ are increased.

These results demonstrate the importance of a precise and free control of the modification time *t*_mod_ and the capabilities of a reaction time *t*_reac_ under low field conditions. Modification times in PTR-MS and HiKE-IMS are in the range of a few hundred microseconds and depend on the modification field. Additional reaction times do not exist. Thus, in these systems, P0 would completely fragment to P4 and the detection of P1 and P3 would not be possible.

### Global Trends

**Figure 10:**
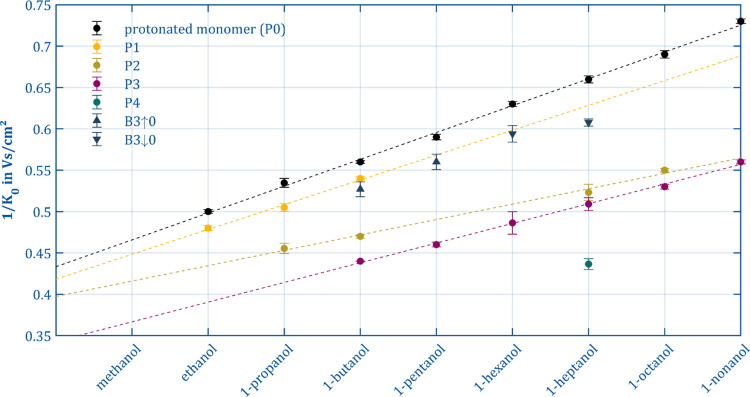
Inverse reduced ion mobilities with 0.95 confidence interval for the precursor (P0, [ROH]H^+^ black) and the product ions P1 (RO^+^, yellow), P2 (R^+^, moss green), P3 ([R – H_2_]^+^, purple), and P4 (teal) and the reaction products B3↑ and B3↓ (dark blue). The measurement parameter can be found in the Supporting Information.

The analysis of 1-butanol and 1-heptanol has demonstrated that information about the ion species present in the ion mobility spectrum recorded in IMS mode can be obtained simply by varying *E*_mod_/*N*, *t*_mod_, and *t*_reac_ in ion manipulation mode. The above investigations have in common that they focus on the product ions of only one selected precursor ion, here, the protonated monomer. Another approach is to study global trends within a substance class.^26^ This approach is adopted in Figure 10, where the inverse reduced mobilities of the protonated monomers (black) acting as the precursor ions and the product ions occurring in ion manipulation mode of 8 primary alcohols with a carbon number *n*_c_ ranging from 2 (ethanol) to 9 (nonanol) are plotted. Unfortunately, the characterization of methanol (*n*_c_ = 1) was not possible as our method of ionization produces no response for this compound. Scaling the horizontal axis in Figure 10 to *n*_c_ = 0 is nevertheless useful, as the intersection with the vertical axis is an important parameter. As discussed in detail above, the product ions appear at different operating parameters depending on the selected precursor ion. Consequently, the data shown in Figure 10 were collected at different modification field strengths, modification times, and reaction times in order to obtain clear and well-separated peaks for the respective ions. These three parameters influence the ratios of the intensities between the product ions but do not affect the ion mobility measurement. The respective measurement parameters can be found in Table S1 in the Supporting Information. The carbon number *n*_c_ is used in the following as a synonym for the protonated alcohols (monomers) serving as precursors. For the sake of simplicity, the resulting product ions are also indexed with the chain length *n*_c_ of the respective precursor, although these could have shorter chains due to fragmentation processes.

The method of representation chosen in Figure 10 simplifies the identification of trends. Furthermore, ions are assigned to the seven collections P0–P4 and the reaction products B3↑ and B3↓ and highlighted in color. According to the results presented by Karpas and Hariharan et al.,^52,53^ a nearly linear relationship between the inverse ion mobility 1/*K*_0_ and the carbon number of the precursor P0 can be expected. Indeed, our data support these findings. The inverse mobility can therefore be described according to eq 2 as a function of the chain length *n*_c_ with the offset fg and the slope *c*.
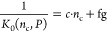
2

Offset and slope are properties of a group *P* of ions; thus, it is reasonable to search for similar relationships in the collection of the product ions. Indeed, the mobilities of the ions of the collection P1 (RO^+^, blue), P2 (R^+^, green), and P3 ([R – H_2_]^+^, orange) are also arranged on a straight line. Analogue to P0, these might be a collection of ions within which the carbon number is successively reduced. The results and goodness of the fits are summarized in Table 2.

**Table 2:** Goodness Adjusted *R*^2^, Slope *c*, and Offset fg of the Linear Fits Describing the Inverse Ion Mobility of the Cluster P0, P1, P2, and P3

cluster	assumed species	slope *c*		offset fg		adj. *R*^2^
		mV s/cm^2^	relative to P0	V s/cm^2^	relative to P0	
P0	[ROH]H^+^	32.1 ± 1.9	1	0.436 ± 0.011	1	0.998
P1	RO^+^	30.0 ± 37.4	0.92	0.418 ± 0.116	0.97	0.981
P2	R^+^	18.6 ± 4.8	0.57	0.397 ± 0.028	0.92	0.989
P3	[R – H_2_]^+^	23.7 ± 2.9	0.73	0.344 ± 0.019	0.79	0.997

The offset fg provides information about the functional group of this cluster of ions. If two clusters have the same offset, then the ions of this cluster should also have the same functional group as the trends will intersect at a carbon chain length of 0. The slope can be understood as a measure of the correlation between *n*_c_ and 1/*K*_0_. According to this, a high slope means that the influence of the carbon chain *R* is high. As stated in Table 2, P0 and P1 show an almost identical slope, while the mobility of P2 and P3 shows a significantly lower slope. This might be attributed to the changed conditions in the localization of the charge. The charge of the ions in the P0 cluster are probably localized at the OH group. If the OH group is omitted, delocalization of the charge and thus a change in the correlation c could be the result. Thus, measuring the slope gives insight into the structural changes of the ion species.

Besides the cluster properties fg and *c*, Figure 10 reveals some other interesting phenomena. Frist, P3 seems to occur only for alcohols with *n*_c_ ≥ 4 and P1 only for *n*_c_ ≤ 4. The latter could be due to the low difference in the ion mobility of P1 and P0 for small alcohols. Thus, the effective temperatures of P0 and P1 are nearly the same. For larger alcohols, the difference is higher, leading to a jump in the effective temperature when P0 fragments to P1. Thus, P1 might immediately fragment further. A second explanation could be the reaction with water. P1 seems to be instable for larger alcohols where the reaction rate increases with the chain length, as can be seen for 1-butanol and 1-pentanol in Figure S1 in the Supporting Information. Thus, for large alcohols, P1 might react directly to P3 after they are formed. The second interesting phenomenon is the reaction with neutral water for larger alcohols. While in case of 1-butanol and 1-pentanol, P3 is the reaction educt, and for 1-hexanol and 1-heptanol, P3 is the reaction product (Figure S1). A first hint could be the calculated enthalpy of fragmentation published by Shokri et al.,^26^ which seem to increase by roughly 25% between a carbon number of 6 and 7 and is constant before. Thus, a change in the fragmentation behavior could be attributed to a more fundamental structural change. Third, there is a gap in the occurrence of P2, which is present only for *n*_c_ = 3 – 4 and *n*_c_ = 7 – 8 but not for 1-pentanol and 1-hexanol. A possible explanation could be a fast reaction removing P2 in favor of one of the other products e.g., by hydrogen abstraction, thus preventing the detection of P2 for 1-pentanol and 1-hexanol. For larger alcohols, P2 is not the carbocation, as shown in Figure 9. Instead, it is the reaction product of P4 and neutral molecules. Here, again, a high reaction rate could be the reason for P4 to be undetectable for 1-octanol and 1-nonanol. This is in good agreement with the increasing rate coefficients for larger alcohols reported by Španěl et al.^48^ We believe that some of the effects, such as the gap in P2, the missing P1 for larger alcohols and the changing reaction product of B3↑ and B3↓, might be due to the limited water vapor concentration range in the drift gas from 40 to 540 ppb_v_. Thus, a better control of the water vapor content could reveal interesting new information. However, this is beyond the scope of this work. Overall, we were able to give a reasonable explanation of most of the product ions, which is summarized again in Table 3.

**Table 3:** Reduced Mobilities *K*_0_ of Protonated Alcohols (P0) and Their Product Ions RO^+^ (P1), R^+^ (P2), and [R – H_2_]^+^ (P3)^a^

compound	molecule structure	*K*_0_ in cm^2^/Vs (precursor)	*K*_0_ in cm^2^/V s (product)				
		P0	P1	P2	P3	P4	
ethanol	C_2_H_6_O	2.00	2.08				
1-propanol	C_3_H_8_O	1.87	1.98	2.19			
1-butanol	C_4_H_10_O	1.79	1.85	2.13	2.27		B3↑
1-pentanol	C_5_H_12_O	1.69			2.17		B3↑
1-hexanol	C_6_H_14_O	1.59			2.06		B3↓
1-heptanol	C_7_H_16_O	1.52		1.91	1.96	2.22	B3↓, B4↑
1-octanol	C_8_H_18_O	1.45		1.82	1.89		B3↓
1-nonanol	C_9_H_20_O	1.37			1.79		

a Furthermore, the occurrence of the reaction products B3↑ and B3↓ is stated.

## Conclusions

In this work, the potential of an IMS with tandem drift regions and an integrated ion manipulator as an extension to the already published FAT-IMS is demonstrated and its utility is shown by studying the fragmentation of eight primary alcohols. The control over the applied energy, the modification time, and the capability to perform reactions with neutral molecules at atmospheric pressure through a storing step offer a wide range of analytical possibilities in only one instrument. Thus, the methods presented here are comparable to the concept of a triple quadrupole MS applied to an IMS. In addition to ion mobility, it is possible to determine further structural and chemical information based on differential mobility, the occurrence, and identity of fragments or chemical reactions with neutral molecules. In application, this could be used for a much more solid characterization and ultimately a drastic reduction of false positive results, for example, in security-related applications where a fast and reliable response is crucial.

However, the potential of the ion manipulator stage has not yet been exhausted. As first results suggest, a reaction with neutral water molecules occurs in the second drift region. To enable further investigations, it is necessary to be able to vary the water concentration in the drift gas in a wider range. Moreover, it is conceivable to introduce humidified air localized directly into the ion manipulator stage, thus enabling investigating the influence of water or other modifiers on the fragmentation and subsequent reactions more precisely, thanks to the adjustable reaction times. This would also make it possible to implement more complex measurement algorithms. For example, precursor ions could be fragmented in the ion manipulator, the products exposed to the modifier for a certain time and then analyzed again in the high electric fields of the ion manipulator. As already explained, coupling the IMS to a mass spectrometer is indispensable for a reliable identification of the product ions for fundamental studies. Nevertheless, it could be shown in this work that the experiments reveal a whole series of interesting observations, proving that an IMS with tandem drift regions and an integrated ion manipulator is a powerful tool for field applications with improved analytical performance compared to classical drift tube IMS.

## Supporting Information Available

Ion mobility spectra of 1-butanol, 1-pentanol, 1-hexanol, and 1-heptanol as mentioned in the text and a brief description of the measurement and measurement parameters utilized for the results (PDF)
