# Supplementary material for: Highly Efficient Ion Manipulator for Tandem Ion Mobility Spectrometry: Exploring a Versatile Technique by a Study of Primary Alcohols
Source: Anal Chem. 2023 Apr 24;95(18):7158–69. doi: 10.1021/acs.analchem.2c05483 (PMC10173250; doi:10.1021/acs.analchem.2c05483)
Supplement: Supplementary file 1 — ac2c05483_si_001.pdf [file ac2c05483_si_001.pdf]

Supporting Information

A Highly Efficient Ion Manipulator for Tandem Ion Mobility Spectrometry – Exploring a Versatile Technique by a Study of Primary Alcohols.

Alexander Bohnhorst[1,2*], Anne Zygmanowski[1], Yu Yin[1], Ansgar T. Kirk[1,2], Stefan Zimmermann[1]

1: Leibniz University Hannover, Institute of Electrical Engineering and Measurement Technology, Department of Sensors and Measurement Technology, 30167 Hannover, Germany

2: ACKISION GmbH, Appelstr. 9A, 30167 Hannover, Germany

[*]Email: bohnhorst@geml.uni-hannover.de

Table of Content

Reaction Behavior for 1-Butanol, 1-Pentanol, 1-Hexanol and 1-Heptanol 2

Measurement Parameters for Figure 10 3

Reaction Behavior for 1-Butanol, 1-Pentanol, 1-Hexanol and 1-Heptanol

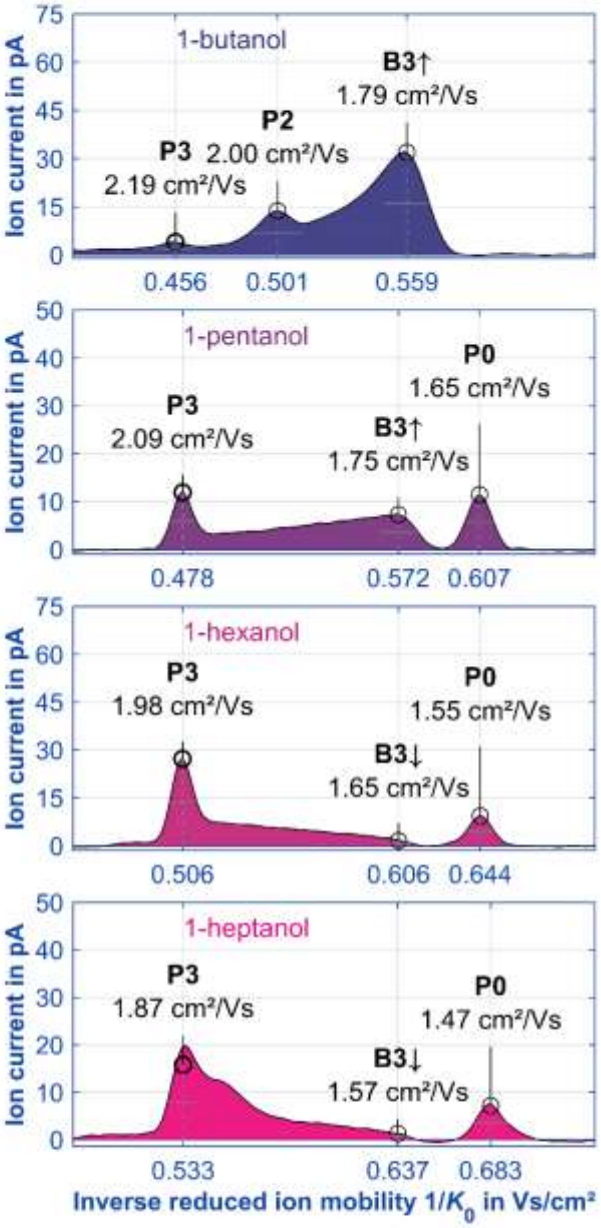

Figure S1: Spectra of 1-butanol at 120 Td in the ion manipulation-mode and 1-pentanol, 1-hexanol and 1-heptanol at 100 Td in the ion manipulator with a water concentration of 540 ppb in the drift gas. The reaction time t_{reac} increases from 2.6 ms for 1-pentanol to 3.4 ms for 1-heptanol as the drift time in the second drift region is increased due to the lower ion mobility of the ions with higher n_c. Furthermore, in case of 1-butanol the drift field in the second drift region is increased by 50 % to reduce the reaction time to 1.6 ms. Otherwise, P3 could not be observed at high humidity. 1-butanol and 1-pentanol show different reaction pattern when compared to 1-hexanol and 1-heptanol, as the stable product changes. In case of 1-butanol and 1-pentanol P3 seems to be the reaction educt while at 1-hexanol and 1-heptanol P3 is the reaction product. (E_{D2}/N = 3 - 4.5 Td, n_{mod} = 5, t_{mod} = 2.5 µs, $t_{reac} = 1.6 - 3.4\ ms$, T = 298 K, P = 1008 mbar)

To further study the nature of the reactions in the drift region, the product ions for the primary alcohols of carbon number 4 to 7 are shown in Figure. Alcohols with a carbon number of 3 and less show no reaction products formed in the drift region, while for alcohols with a carbon number of 8 and higher the reaction rates are too high and only peaks with Gaussian shape can be detected. The direction of the reaction reverses between 1-pentanol and 1-hexanol. While the slope of the baseline of the 1-pentanol indicates that P3 is still the reaction educt, the reverse slope of the baseline for the 1-hexanol indicates that P3 is the reaction product. An explanation for this is not possible without mass analysis. Mainly due to electrical reasons, coupling the IMS with tandem drift regions and integrated ion manipulator is difficult, especially when the MS inlet is at ground potential as this implies the ion

manipulator and thus the high voltage driver electronics must be operated at high potential. We are working on such a solution, but cannot provide mass data yet. However, a possible hypothesis could be that the reaction product and educt are in equilibrium when leaving the ion manipulator and only in the second drift region, with the participation of water, the reaction shifts in one direction or the other. This tipping point seems to be dependent on the carbon number and the water content

Measurement Parameters for Figure 10

Table S1: Measurement parameters used for the data points shown in Figure 10.

Compound	Product ion	E_{mod}/N in Td	t_{mod} in µs	n_{mod}	t_{reac} in ms	Water content in ppb
1-Ethanol	P1	105	5	10	3.4	540
1-Propanol	P1, P2	92	5	10	3.5	540
1-Butanol	P1, P2, P3	106	2.5	5	2.7	40
1-Pentanol	B3↑	120	2.5	5	2.7	40
	P3, B3↑	100	5	10	3.1	540
1-Hexanol	P3	120	1	2	2.9	540
	B3↓	120	0.5	1	7.7	540
1-Heptanol	P2, P3	120	7.5	15	3.9	540
	B3↓	120	1	2	3.2	540
	P4, B4↑	120	17.5	35	1.6	230
1-Octanol	P2, P3	120	5	10	3.5	540
1-Nonanol	P3	120	5	10	3.7	540
